# Real-World Experience with Nintedanib in a Progressive Pulmonary Fibrosis Cohort

**DOI:** 10.3390/medicina62061187

**Published:** 2026-06-18

**Authors:** Vanesa Vicens-Zygmunt, Jaume Bordas-Martínez, Miriam Muñoz-Bolaño, João Carmezim, Ana Belén Llanos-González, Adrià Domingo-Carnice, Dolores Rodríguez-Cumplido, Guadalupe Bermudo-Peloche, Cristian Tebé-Cordomí, Judith Peñafiel, Roser Llop-Rius, Maria Molina-Molina

**Affiliations:** 1 Interstitial Lung Disease (ILD) Unit, Pulmonology Department, University Hospital of Bellvitge, Institute of Research of Bellvitge (DIBELL), University of Barcelona (UB), Biomedical Research Network Center Respiratory Diseases (CIBERES), 08907 Barcelona, Spain; 2 Interstitial Lung Disease (ILD) Unit, Pulmonology Department, Hospital General de Granollers, 08402 Granollers, Spain; 3 Pharmacy Department, University Hospital of Bellvitge, 08907 Barcelona, Spain; 4 Biostatistics Unit (UBiDi), Bellvitge Biomedical Research Institute (IDIBELL), 08840 Barcelona, Spain; 5 Interstitial Lung Disease Unit, Pulmonology Department, University Complex Hospital of Canarias, 38320 Santa Cruz de Tenerife, Spain; 6 Clinical Pharmacology Department, University Hospital of Bellvitge, 08907 Barcelona, Spain

**Keywords:** pulmonary fibrosis, antifibrotic, real-world, pulmonary function tests, immunosuppressants, cohort

## Abstract

*Background and Objectives:* Nintedanib is indicated for progressive pulmonary fibrosis (PPF) based on clinical trial results. The primary objectives of this study were to evaluate the effectiveness of nintedanib on forced vital capacity (FVC) and diffusing lung capacity for CO (DLCO) after one year of treatment, and to compare the annual rate of decline (“slope”) for FVC, DLCO and 6 minute walking distance (6MWD) with the year prior to treatment. The secondary objectives were antifibrotic safety, tolerability, adverse events, immunosuppressant use, dyspnoea and survival. *Materials and Methods*: This study was a single-centre, retrospective, observational cohort study that included consecutive patients with PPF treated with nintedanib. *Results*: Fifty-five patients with non-IPF fibrotic ILD initiated nintedanib due to fibrosis progression. Most patients (63.4%) stabilised/improved FVC after 1 year of treatment, and 82.5% stabilised/improved DLCO. The “slope” of FVC and DLCO was reduced after 1 year of treatment compared to the year before initiation, although the difference was not statistically significant: FVC slope was +0.61% in the year after initiation vs. −2.3% in the year prior (mean change: 2.94%, 95%CI [−4.74, 10.62]); DLCO slope was −3.8% after treatment vs. −7% before initiation (mean change: 3.24%, 95%CI [−7.43, 13.92]). Dyspnoea improved in 23.2% of patients. A reduction in immunosuppressant use was observed after nintedanib initiation. Forty-one patients (74.5%) experienced at least one side effect: diarrhoea (60%), hepatotoxicity (23.6%), or asthenia (12.7%). Fifteen patients required permanent or temporary treatment discontinuation. *Conclusions*: In our real-world PPF cohort, most patients showed FVC and/or DLCO stabilisation or improvement after one year of nintedanib treatment. A non-significant reduction in the rate of FVC decline after one year of treatment was also observed, as was a reduction in symptom severity in our real-life PPF cohort.

## 1. Introduction

Progressive pulmonary fibrosis (PPF) involves different fibrosing interstitial lung diseases (ILD), other than idiopathic pulmonary fibrosis (IPF). These entities share common clinical, physiological, and radiological characteristics with a poor prognosis despite the initial therapy received—generally corticosteroids and/or immunomodulators, depending on the type of ILD [1,2,3,4,5,6,7]. It is known that PPF shares common pathogenic fibrosing pathways with IPF related to fibrosis progression [1,2,3,4,5,6,7]. A recent official ATS/ERS/JRS/ALAT Clinical Practice Guideline [1] provides a definition of PPF.

Nintedanib, an intracellular inhibitor of tyrosine kinases, has been approved for the treatment of fibrosis associated with systemic sclerosis (SSc-ILD) (SENSCIS phase 3 trial, NCT02597933) [8,9,10] and in PPF based on the double-blind, randomised, placebo-controlled INBUILD phase 3 clinical trial (NCT02999178) [11,12], which showed benefits in reducing the annual rate of decline in forced vital capacity (FVC). However, a post hoc analysis of the INBUILD trial suggested different responses in FVC decline depending on the ILD entity [13]. Furthermore, there is unclear evidence about the magnitude of reduction in disease progression, since the clinical trials did not capture the severity of FVC and diffusing lung capacity for carbon monoxide (DLCO) slope decline before starting drug therapy. On the other hand, there is scarce real-world experience published in PPF, and clinical practice does not always maintain the same treatment criteria as in clinical trials. The aim of this retrospective study was to evaluate the effectiveness of nintedanib in PPF in real-world practice within a Spanish cohort.

## 2. Materials and Methods

This was a single-centre, retrospective, observational, longitudinal cohort study conducted at our University Hospital. The aims of this study were to assess the effectiveness and safety of nintedanib in patients with progressive pulmonary fibrosis (PPF). The data were collected from 55 consecutive patients from the interstitial lung disease (ILD) Unit who initiated off-label nintedanib treatment between 2015 and 2020. Inclusion criteria included a fibrotic ILD diagnosis made according to the ATS/ERS criteria [14] by the multidisciplinary committee.

Patients initiated antifibrotic treatment when they met the criteria for PPF despite prior attempted treatment. PPF was defined as either a ≥5% decline in percent predicted FVC, a ≥10% decline in percent predicted DLCO with significant symptomatic deterioration not due to other causes, or worsening of fibrotic radiological signs (e.g., septal thickening, traction bronchiectasis, and/or honeycombing) in the thorax high-resolution computed tomography (HRCT). The antifibrotic treatment was prescribed as an off-label use and approved by the medication committee in special situations (CMSE in Spanish acronym) from our centre, due to the period of treatment previous to the results of the INBUILD trial. The dose of nintedanib at the beginning of treatment is always 100 mg/12 h and it is usually increased to 150 mg/12 h after one week of treatment for all patients. In our clinical practice, we reduce the dose (from 150 to 100 mg/12 h) with diet recommendations in the majority of patients before discontinuation.

Demographics were obtained at diagnosis. Comorbidities and clinical data were either reported by the patient during the initial assessment or during the follow-up. Demographic variables collected were: age, gender, smoking status, clinical data (dyspnoea, obtained by mMRC (modified Medical Research Council scale) during the medical visits), family history, telomere length (when available), comorbidities (arterial and pulmonary hypertension, cardiopathy, gastroesophageal reflux, diabetes, dyslipidaemia, pulmonary thromboembolism, apnoea, emphysema, neoplasia), radiological pattern on thorax HRCT, and treatments (ruxolitinib and immunosuppressants [corticosteroids, mycophenolate, rituximab, leflunomide, azathioprine, sirolimus, hydroxychloroquine, abatacept, methotrexate, tocilizumab, adalimumab]).

The primary objective of this study were: (1) toevaluate the effectiveness of nintedanib on the %FVC predicted and %DLCO predicted after 1 year of treatment, with Failure defined as a decrease in more than 5% in %FVC and/or ≥10% of DLCO predicted at 12 months, and Success (stability/improvement**)** as stability of %FVC predicted (increase or decrease up to 5%) and/or %DLCO (increase or decrease up to 10%) or improvement (increase in FVC greater than 5% and/or increase in DLCO ≥ 10%); (2) to evaluate the annual rate of decline (slope) of FVC, DLCO, and 6 minute walking distance (6MWD), performing a comparative analysis between the year before the antifibrotic treatment and the year after its prescription.

The 3 time-points for pulmonary function data collection included: at treatment initiation, 1 year before, and 1 year after medication. Of the 55 patients, 36 had data from all 3 time-points for FVC and DLCO analyses, and 28 patients had all 3 time-points for 6MWD analyses. The remaining patients (19 patients for FVC/DLCO and 27 patients for 6 MWD) were excluded from the slope analysis. This is a key limitation; however, we preferred to analyse the data by discarding missing values rather than imputing them, in order to avoid uncertain results.

The secondary objectives were to evaluate the effect of nintedanib on walking distance (metres) and oxygen requirements on exertion, dyspnoea, survival, concomitant immunosuppressant use, antifibrotic adverse events, drug safety, tolerability, and treatment change or discontinuations. Dyspnoea and oxygen requirements were assessed at three time-points (1 year before, at the beginning of nintedanib treatment, and 1 year after its initiation). Survival of all treated patients was analysed considering the time from diagnosis (first visit in the ILD-Unit) until 2024 (last revision of survival). Causes of mortality were described. Concomitant immunosuppressant use was evaluated the year before and after nintedanib initiation. A *p*-value of <0.05 was considered statistically significant. This study was approved by the Ethics Committee of University Hospital of Bellvitge (reference codes PR307/16 and EOM034/21). Authors from this manuscript hold the posting rights of the entire content. The content of this manuscript is entirely original, and artificial intelligence was not used in its preparation.

### Statistical Analysis

For the primary analysis (effectiveness and rate of decline of FVC and DLCO after one year of treatment), the chi-square test was used. For the secondary analysis, the change in each parameter was evaluated through the comparison of means using Student’s *t*-test if the distribution of the variable was normal or a Wilcoxon signed-rank test if the distribution was not normal. For the rate of decline of 6MWD, a Wilcoxon signed-rank paired test was used. For descriptive analysis, frequency (*n*, %) was used for categorical variables; mean and standard deviation (SD) for normally distributed continuous variables; and median and interquartile range (IQR) for non-normally distributed variables.

A description of nintedanib toxicity and the need for dose reduction or treatment discontinuation was also made. In addition, the time from diagnosis to nintedanib prescription was also analysed to evaluate treatment delay. The mean survival time and mortality analyses (Kaplan–Meier) included the entire study population (*n* = 55) from ILD diagnosis until 2024 (last revision of survival). Whenever possible, point estimates were accompanied by a 95% confidence interval (CI) (*p*-value < 0.05 was considered statistically significant). Statistical analyses were performed with R (version 4.2.1 or higher).

## 3. Results

### 3.1. Demographic Characteristics

Between 2015 and 2020, 55 patients with PPF initiated nintedanib due to fibrotic progression. Demographic data and comorbidities are included in Table 1. From this cohort, dyslipidaemia and arterial hypertension, followed by gastroesophageal reflux and pulmonary arterial hypertension (PAH), were the most frequent comorbidities. PAH was present in 16 patients (29%). Concomitant medication, including immunosuppressants to treat specific diseases (CTD-ILD, HP, idiopathic NSIP, uILD…) and ruxolitinib for myelodysplastic syndrome and idiopathic myelofibrosis, was present in 44 patients (80%).

**Figure 1:**
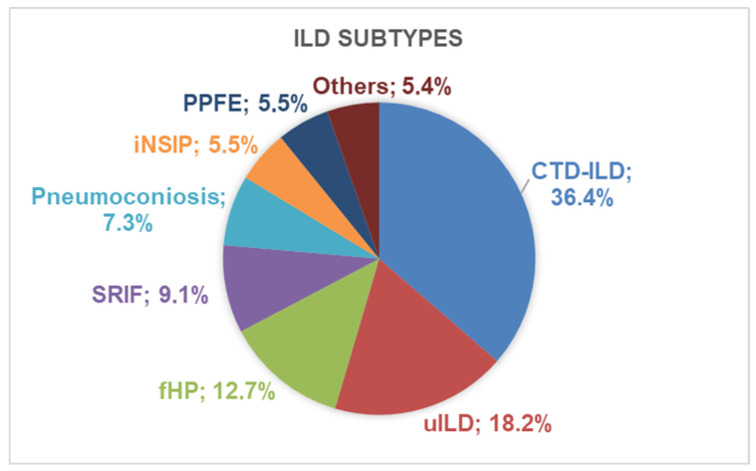
Interstitial lung disease (ILD) subtypes. Connective tissue disease (CTD), unclassifiable ILD (uILD), fibrotic Hypersensitivity Pneumonitis (fHP), smoking-related interstitial fibrosis (SRIF), Pneumoconiosis (silicosis, asbestosis), Idiopathic non-specific interstitial pneumonia (iNSIP), Pleuro-parenchymal fibroelastosis (PPFE). Others: sarcoidosis (1.8%), post-Acute Respiratory Distress Syndrome (ARDS) in a patient with myelofibrosis (1.8%) and combined pulmonary fibrosis-emphysema (CPFE, 1.8%).

The main diagnoses are represented in Figure 1, and the most prevalent were CTD-ILD (36.4%), uILD (18.2%), fHP (12.7%), and SRIF (9.1%). The specific CTD-ILD diagnoses were rheumatoid arthritis (n 8), systemic sclerosis (n 9), antisynthetase syndrome (n 1), vasculitis (n 1), and interstitial pneumonia with autoimmune features (IPAF, n 1).

The median time from the beginning of respiratory symptoms to nintedanib prescription was 47.3 months [IQR 27.1; 68.3]. The median time from diagnosis to nintedanib prescription was 21.2 months [IQR 7.8; 44.3].

Pulmonary function test data at the beginning of nintedanib treatment are described in Table 1 (data from 49 patients for FVC and 45 patients for DLCO). Patients presented with FVC > 40% and DLCO > 25% at the time of nintedanib initiation. The median FVC and DLCO before starting nintedanib were 82% of predicted [IQR 70.8; 99.3] and 49% of predicted [IQR 40.5; 57.6], respectively (Table 2). The median distance travelled in the 6MWT (6 min walking test) before the beginning of nintedanib was 411.0 metres [IQR 358.3; 485.5] (Table 2). Regarding supplemental oxygen during exertion, requirements became progressively more frequent, independently of nintedanib treatment.

### 3.2. Effect of Nintedanib on Pulmonary Function

The primary analyses regarding the success or failure of nintedanib treatment one year after its initiation are presented in Table 2 and show the following results: FVC stabilisation or improvement was observed in 63.4% of patients (“successful response”) after 1 year of nintedanib treatment, and 36.6% continued to worsen. Regarding % predicted DLCO, 82.5% of patients stabilised or improved (“successful response”) after 1 year of nintedanib treatment, and 17.5% worsened. Immunosuppressant treatment did not show any influence on these results (Table 2). However, the heterogeneity of ILD types must be kept in mind, as some of them may exhibit periods of relative stability independently of therapy.

The primary analyses regarding the rate of decline of % predicted FVC the year after nintedanib initiation showed a reduction compared to the previous year, though it was not statistically significant. FVC increased by +0.61% the year after nintedanib initiation, while it had declined by 2.3% the year before starting, representing a mean change of 2.94% (95% CI [−4.74, 10.62]), *p*-value 0.442 (Table 3). Additionally, the decline of % predicted DLCO after nintedanib initiation was not statistically significantly lower than the previous year: DLCO decreased by 7% the year before beginning nintedanib and decreased by 3.76% the year after its initiation, with a mean change of 3.24% (95% CI [−7.43, 13.92]), *p*-value 0.541 (Table 3).

Concerning the walked distance on the 6MWT, the analysis exhibited a decline in walking distance (metres) despite the antifibrotic treatment (Table 3). 6MWD did not decline before beginning nintedanib but decreased by 8.85% the year after its starting, with a % of change of −8.98% (95% CI [−19.31, 1.35]), *p*-value 0.086.

### 3.3. Secondary Outcomes: Symptoms, Mortality/Lung Transplant, Mean Survival Time, and Immunosuppressant Reduction

**Figure 2:**
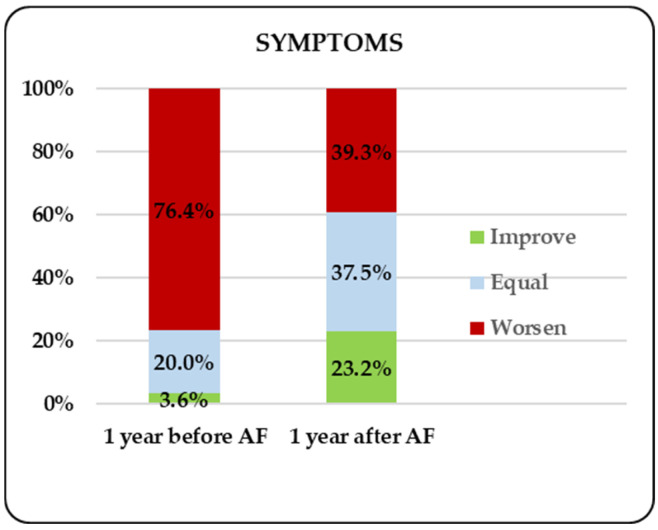
Symptom change (dyspnoea) 1 year before and 1 year after nintedanib treatment. Twenty-three point two percent (23.2%) of patients improved dyspnoea after 1 year of nintedanib treatment, 37.5% perceived the same symptoms and 39.3% worsened compared with the year before its initiation, when 76.4% of patients got worse. Of the 39.3% of patients whose symptoms worsened, 5.4% required lung transplant (n 3) and 12.7% died (n 7) during the first year of treatment.

Regarding symptoms (Figure 2), 23.2% of patients reported improved dyspnoea after 1 year of nintedanib treatment, 37.5% perceived the same symptoms, and 39.3% worsened. This is compared to the 76.4% of patients who reported worsening symptoms the year before its initiation. Of the patients who reported worsening symptoms (39.3%), three required lung transplant (5.4%) and seven patients (12.7%) died during the first year of treatment.

**Figure 3:**
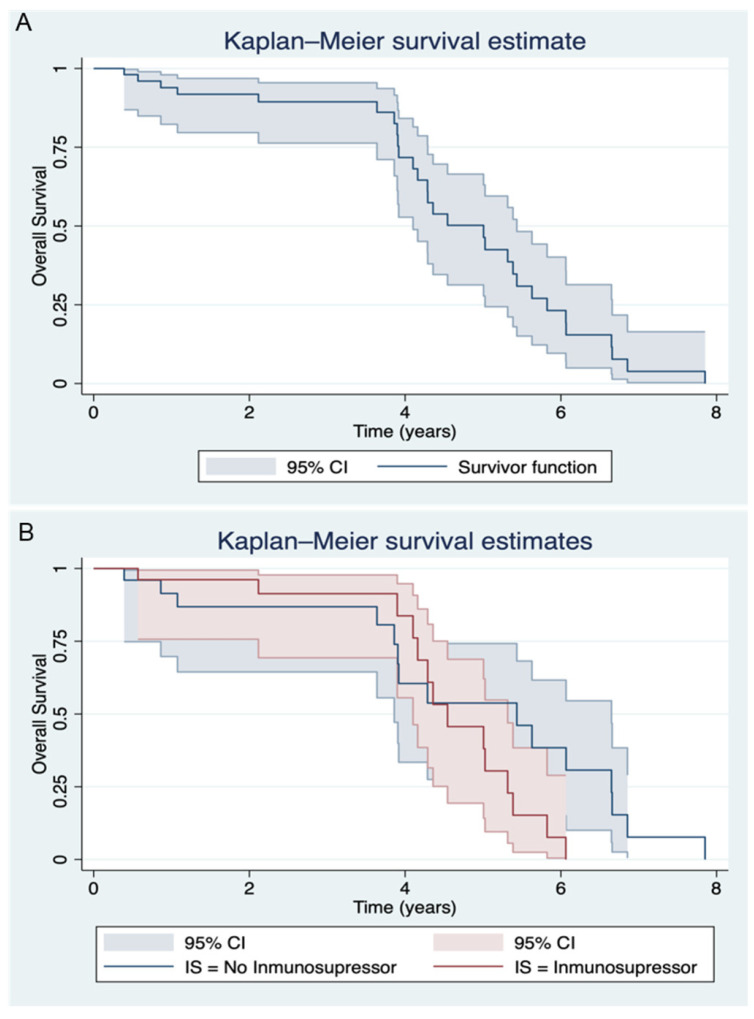
Mortality in patients treated with nintedanib with/without immunosuppressant (IS) treatment. (**A**). Kaplan–Meier from overall patients from the cohort (n 55). (**B**). Patients with IS treatment (taking into account patients treated with all IS and also prednisone ≥ 10 mg/day) and without IS treatment (also taking into account patients treated with prednisone < 10 mg/day). Analysed by Mantel–Cox (logrank test), *p* 0.170. Please check if any symbols are missing here.

The mean survival time was 56.6 months (95% CI 50.2–63.1) from the beginning of nintedanib (Figure 3).

Death during the first year of treatment was related to acute exacerbation (three patients), fibrosis progression (two patients), and neoplasia (two patients). Another 11 patients treated with nintedanib died (20%) and four required lung transplant (7.3%) during the follow-up period of 1.5 to 6 years after nintedanib initiation. Death in these cases was related to acute exacerbation (five patients), fibrosis progression (three patients), and neoplasia (three patients) (Table 4).

The median % predicted for FVC and DLCO for those patients who died during the first year of treatment was 59% [IQR 38; 71] and 39% [IQR 32; 44], respectively. Patients who died during the first year of treatment presented % predicted FVC that was significantly lower at drug initiation (57.8%, SD 25.9) than those patients alive during the first year (80.7%, SD 24.5), *p*-value 0.032. No significant differences were observed in DLCO between patients who died or were alive during the first year of treatment (38.0% (SD 9.1) vs. 49.7% (SD 14.6), *p*-value 0.127.

Concerning mortality, of the 18 patients who died, four showed familial clustering and 12 were treated concomitantly with immunosuppressant drugs (one systemic sclerosis, four rheumatoid arthritis, two uILD, one idiopathic fibrosing NSIP, two fHP, two SRIF); but the Kaplan–Meier survival analysis did not find differences in mortality depending on immunosuppression treatment, *p*-value 0.170 (Figure 3). However, more fibrosis progression and acute exacerbations were observed in patients receiving immunosuppressant treatment (Table 4).

Finally, a reduction in corticosteroid dose and a reduction in immunosuppressant use was observed the year after nintedanib initiation compared to the year prior to its starting (Table 5).

### 3.4. Safety

A total of 41 patients (74.5%) experienced at least one adverse event, most of which were mild; 15 patients (27.3%) had one adverse event and 26 patients (47.3%) had two or more. Of the 67 adverse reactions, the most frequent were diarrhoea (60%), hepatotoxicity (23.6%), and asthenia (12.7%). Fifteen patients (27.3%) had an adverse event leading to permanent or temporary treatment discontinuation, with diarrhoea (93.3%), asthenia (26.7%), hepatotoxicity (20%), hyporexia (13.3%), and weight loss (13.3%) being the most frequent causes. Thirteen patients from the group who discontinued nintedanib switched to pirfenidone. Eleven patients (84.6%) experienced at least one adverse effect with the new treatment. The most frequent adverse reactions were gastrointestinal and photosensibility (23.1%).

Concomitant use of immunosuppressant drugs was associated with a higher frequency of adverse events like diarrhoea (63.2%) and hepatotoxicity (26.3%) compared to nintedanib alone (35.3% and 11.8% respectively). The change in antifibrotic treatment because of adverse events had no significant impact on the overall results of % FVC and DLCO predicted.

## 4. Discussion

PPF affects different non-IPF ILDs, often with a behaviour similar to IPF and a poor prognosis. The aim of this study was to evaluate the behaviour of FVC and DLCO in patients who initiated nintedanib as an off-label treatment when they met INBUILD criteria to identify PPF [11], and to analyse dyspnoea, side effects of the treatment, and mortality. It is important to note that this study lacks a control group, so the analyses were performed within the cohort itself (pre/post treatment), and the observed changes may therefore not be attributable solely to nintedanib treatment.

The majority of patients from our cohort stabilised/improved FVC and DLCO after one year of treatment, although the mean rate of FVC and DLCO decline was reduced with no statistically significant difference. Considering the heterogeneity of different types of ILD, the observed stabilisation/improvement cannot be attributed solely to nintedanib treatment, as some ILDs exhibit periods of relative stability regardless of therapy. An exploratory subgroup analysis in larger cohorts with a control group could clarify this point. However, Cameli P et al. also observed a significant reduction in the rate of FVC decline compared to pre-treatment data, without observing a difference in DLCO decline rate in a cohort of 30 non-IPF PPF patients from Siena [15]. In addition, a decrease in dyspnoea was also reported after 1 year of nintedanib treatment. However, the mean 6MWD decreased progressively over time with increased requirements for supplemental oxygen on exertion despite nintedanib treatment. The decrease in metres walked in the 6MWT could be associated with the natural evolution of progressive patients adapting to a lower exercise capacity, comorbidities such as pulmonary hypertension and higher oxygen requirements on exertion. It is important to highlight this, as it differs from the benefits observed in pulmonary function tests, demonstrating a potential gap between physiological and functional benefits.

Interestingly, it is suggested that PPF patients may show a different response to nintedanib depending on the type of fibrotic ILD [16]. Our cohort is too small to sub-analyse these differences; however, it would be of interest to analyse data from larger cohorts, considering the different lung function trajectories from different ILD subtypes as described by Oldham JM and colleagues [17], and considering the influence of the % FVC previous to nintedanib treatment as proposed by Maher TM et al. [18]. In this sense, patients who died during the first year in our cohort had poorer FVC than those alive.

Furthermore, the proportion of patients for each ILD who were classified as PPF was similar to previous studies, with CTD-ILD (36.4%), uILD (18.2%), and fHP (12.7%) being the most frequent in our cohort [19,20].

Initiation of antifibrotic treatment is often delayed more than one year from final diagnosis, and a longer delay is observed when time from symptom onset is considered [21,22,23,24]. As described in the literature [11], we observed a delay from diagnosis in the prescription of antifibrotic treatment, around 21.2 months [IQR 7.8; 44.3], observing 12.7% of deaths during the first year of treatment in patients with poorer % predicted FVC (*p* = 0.032). However, patients in this PPF cohort may have been progressing previously, which differs from the active monitoring for PPF in our current clinical practice. Mortality and lung transplant rates during the first year of treatment were higher than in the INBUILD trial [12] but lower than in a real-world Japanese non-IPF cohort (29.1%) [25]. However, Niitsu’s cohort [25] presented a slightly older age than our cohort (69.5 [63.25; 74]). Another cohort in Siena showed a 7-year mortality of 26.7%, and patients showed slightly better pulmonary function tests than our cohort at baseline [15]. According to other authors, baseline FVC less than 70% of predicted value and baseline DLCO less than 50% of predicted value in the year prior to initiation of antifibrotic treatment have been associated with a worse outcome [15], and patients who died in our cohort met these criteria, also considering that treatment was prescribed off-label before of INBUILD results.

Interestingly, although there is a small number of patients in our cohort, we observed that acute exacerbations, fibrosis progression, and neoplasia were the most frequent causes of death, with exacerbations and fibrosis progression being increased in patients receiving immunosuppressant treatment (IS) compared to those without it. While IS could stabilise the underlying ILD, preferentially in CTD-ILD, it could have a deleterious effect in other ILD entities that have already triggered pathogenic fibrosing pathways with less inflammatory substrate [6,7]. This may be associated with an increased risk of death, possibly in cases with telomere shortening, as occurred in the PANTHER-IPF clinical trial (NCT00650091) [26]. Unfortunately, no data on telomere length were available for the analysed patients. This is only a possible interpretation, as patients treated with immunosuppressants usually tend to have a more severe clinical condition or a more aggressive form of the disease in clinical practice.

Otherwise, we observed a reduction in corticosteroid dose and IS treatment use during the first year of the antifibrotic prescription. To our knowledge, this study is the first to evaluate the reduction in immunosuppressant use the year after antifibrotic initiation. This result could shed important light on the possibility of reducing acute exacerbation and secondary fibrosis progression by reducing IS treatment in patients with non-IPF fibrosing ILD treated with antifibrotics, particularly when clinical and pathobiological mechanisms of progression are similar to IPF [6,7], keeping in mind the PANTHER trial [26]. This observation is only a possible hypothesis. In our clinical practice, we reduce immunosuppressants in the majority of patients when we observe improvement (to give them the minimum dose of immunosuppressants that maintains the patient at a stable level with the least adverse events and try to withdraw corticosteroids); however, they are sometimes reduced because of adverse events, and these data were not recorded in this retrospective study.

Regarding drug safety, diarrhoea and hepatotoxicity were the most frequent adverse reactions observed in our study, similar to the reports in the INBUILD trial [11]. The use of carob flour (since 2018) for diarrhoea, and dose reduction or stopping administration of nintedanib for hepatotoxicity, was useful to control these adverse events. Interestingly, Cota E et al. [27] from Portugal reported a 26.7% discontinuation rate and suggested that lower baseline body surface area was a risk factor for nintedanib discontinuation and for early-onset gastrointestinal adverse events. Furthermore, the concomitant administration of immunosuppressant treatment increased diarrhoea and hepatotoxicity in our cohort compared with nintedanib alone without reaching statistical evidence. This observation could be related to an interaction between both treatments or to the underlying disease (mostly CTD). However, the small sample size lacks the statistical power to establish causality, and it could be interesting to explore this hypothesis in larger prospective cohorts. The mechanism leading to diarrhoea in patients treated with nintedanib remains unknown. In our cohort, we observed 11 patients treated with IS ± prednisone with diarrhoea, 13 patients treated with prednisone alone who had diarrhoea and five patients without either IS or corticosteroids who presented with diarrhoea. A previous report in IPF [28] described that the concomitant use of prednisolone with nintedanib reduced diarrhoea in their Japanese cohort. These results differ from ours. We did not include IPF patients in our study. It is possible that in patients with non-IPF progressive pulmonary fibrosis, concomitant use of corticosteroids and/or immunosuppressants may be deleterious, increasing gastrointestinal events. This may occur through direct intestinal mucosal damage or through potentiation of the effects of tyrosine-kinase inhibition, as proposed by Kato M et al [28]. Recently, Tsubouchi K et al. [29] reported a similar proportion of adverse events in PPF patients who received early combination therapy with tacrolimus, prednisolone and nintedanib, observing diarrhoea (67.6%) and hepatic dysfunction (29.4%) comparable with our cohort. In another article, Fujiwara M et al. [30] reported that the concomitant use of prednisolone with nintedanib in a PPF cohort may also be associated with gastrointestinal haemorrhage. These studies in PPF cohorts suggest that the mechanism of diarrhoea in PPF (non-IPF) patients may differ from that in IPF. It would be of interest to evaluate this issue in prospective studies with larger sample sizes.

Although there were few cases of systemic sclerosis in our cohort (nine patients), adverse events were frequently observed, leading to nintedanib discontinuation as was also reported in the SENSCIS trial [9]; however, asthenia and weight loss were also observed in those patients. Indeed, the safety profile of our study is consistent with suspected adverse events reported in the Summary of Product Characteristics (SmPC).

This study has limitations inherent in the retrospective analysis, small sample size, heterogeneity of fibrosing ILD entities, and missing data from function tests during the follow-up or before the antifibrotic treatment. Patients who initiated nintedanib at that time (2015–2020) were probably more severe in terms of fibrosis than currently. It would be relevant to evaluate the beneficial effects and survival of nintedanib in larger PPF cohorts, during longer periods, and to analyse the benefits of early antifibrotic prescription, identifying the profile of patients who would benefit most.

## 5. Conclusions

Most patients treated with nintedanib showed FVC and DLCO stabilisation or improvement after one year. A non-significant reduction in the rate of FVC decline after one year of treatment was also observed, as was a reduction in symptom severity in our real-life PPF cohort.

## Figures and Tables

**Table 1:** Demographic data, comorbidities, immunosuppressant treatment and pulmonary function tests (FVC and DLCO severity) from patients at the beginning of nintedanib treatment.

Demographic Data and Comorbidities	Nintedanib Treatment (n 55)
Age , Media years old (SD) Median years old [Q1; Q3]	63.4 (11.0) 63.0 [59.0; 71.0]
Gender, N (%) Female Male	28 (50.9%) 27 (49.1%)
Smoker, N (%) Never Active Former	26 (47.3%) 3 (5.4%) 26 (47.3%)
Arterial hypertension, N (%)	23 (41.8%)
Diabetes mellitus, N (%)	9 (16.4%)
Dyslipidaemia, N (%)	23 (41.8%)
Gastroesophageal reflux, N (%)	19 (34.5%)
Cardiopathy, N (%)	9 (16.4%)
Pulmonary Arterial Hypertension, N (%) Systemic Sclerosis (SSc) Idiopathic fibrosing NSIP CPFE, sarcoidosis, uILD, fHP, SRIF, silicosis and post-ARDS in a patient with idiopathic myelofibrosis	16 (29%) 7 2 1
Pulmonary embolism, N (%)	3 (5.4%)
Sleep apnoea hypopnea syndrome, N (%)	3 (5.4%)
Neoplasia, N (%)	11 (20%)
Emphysema less than 10% extension, N (%)	13 (23.6%)
CPFE, N (%)	1 (1.8%)
Familial pulmonary fibrosis, N (%) Telomere length < 10%, N (%)	12 (21.8%) 3 (25% from 12 patients)
Immunosuppressant treatment (including corticosteroids), N (%) Corticosteroids treatment, N (%) <15 mg/day Mycophenolate Rituximab Leflunomide Abatacept/Hydroxychloroquine Methotrexate/Tocilizumab/Adalimumab/ Tacrolimus/Sirolimus Ruxolitinib	44 (80%) 40 (72.7%) 34 (61.8%) 13 (23.6%) 9 (16.4%) 4 (7.3%) 2 (3.6%) 1 (1.8%) 2 (3.6%)
FVC severity at the beginning of Nintedanib, N 49 * (%) Normal (≥80%) Mild (70–79%) Moderate (60–69%) Moderate-Severe to Very Severe (<60%)	23 (46.9%) 10 (20.4%) 4 (8.2%) 12 (24.5%)
DLCO severity at the beginning of Nintedanib, N 45 ** (%) Normal (≥80%) Mild (70–80%) Moderate (50–69%) Severe (35–50%) Very severe (<35%)	2 (4.4%) 4 (8.9%) 13 (28.9%) 21 (46.7%) 5 (11.1%)

* Six patients had no FVC at the beginning of treatment. ** 10 patients had no DLCO at the beginning of treatment. Forced vital capacity (FVC), diffusing lung capacity for CO (DLCO), systemic sclerosis (SSc), idiopathic fibrosing non-specific interstitial pneumonia (iNSIP), combined pulmonary fibrosis-emphysema (CPFE), unclassifiable ILD (uILD), fibrosing Hypersensitivity Pneumonitis (fHP), smoking-related interstitial fibrosis (SRIF), Acute Respiratory Distress Syndrome (ARDS).

**Table 2:** Success or failure after 1 year of Nintedanib treatment for FVC and DLCO. IS (Immunosuppressant), +/− (with/without)), + (with).

FVC	Success	Failure
Nintedanib +/− IS (n 41) N (%)	26 (63.4%)	15 (36.6%)
Nintedanib + IS (n 33) N (%)	20 (60.6%)	13 (39.4%)
DLCO	Success	Failure
Nintedanib +/ − IS (n 40) N (%)	33 (82.5%)	7 (17.5%)
Nintedanib + IS (n 31) N (%)	26 (83.9%)	5 (16.1%)

N is adjusted for patients with FVC and DLCO data at least two time-points (before nintedanib treatment and 1 year after its initiation). For FVC, failure was defined as a decrease in more than 5% in FVC at 12 months, and success was defined as stability of FVC (increase or decrease up to 5%) or improvement (increase in FVC greater than 5%). For DLCO, failure was defined as a decrease of ≥10% in DLCO at 12 months, and success was defined as stability of DLCO (increase or decrease up to 10%) or improvement (increase in DLCO ≥ 10%).

**Table 3:** Median of %FVC, %DLCO and 6 minute walking distance (6MWD (metres)) 1 year before the antifibrotic (AF) treatment, at the beginning of AF treatment and 1 year after its initiation. Percentage of change in FVC predicted (% FVC pred), %DLCO pred and walked distance on the 6MWT before and after nintedanib treatment. Evolution graph of %FVC, %DLCO and distance travelled before and after AF initiation. CI (Confidence Interval). Results are presented as Median [Q1; Q3].

	N	1 Year Before Nintedanib	Beginning Nintedanib	% of Change the Year Before	1 Year After Nintedanib	% of Change the First Year After	% of Change (Difference)	CI95%	*p*-Value
%FVC	36 *	83.3 [72.5; 105.3]	82.0 [70.8; 99.3]	−2.33%	81.0 [68.3; 96]	0.61%	2.94%	[−4**.74; 10.62]**	**0.442**
%DLCO	36 *	53 [45.8; 63.1]	49.0 [40.5; 57.6]	−7%	44.5 [39.5; 55]	−3.76%	3.24%	[−7**.43; 13.92]**	**0.541**
6MWD (metres)	28 **	430.5 [372.3; 500.3]	411.0 [358.3; 485.5]	0.13%	395 [353.5; 464]	−8.85%	−8.98%	[−1**9.31; 1.35]**	**0.086**
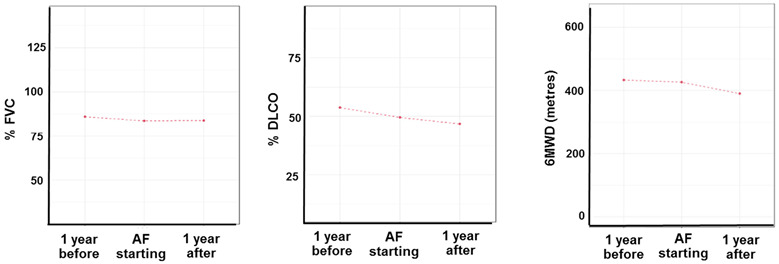									

* 19 patients have no data from the three times. ** 27 patients have no data from the three times.

**Table 4:** Cause of mortality in patients treated with nintedanib with/without immunosuppressant (IS) treatment. Fibrosis progression and acute exacerbations (AEx) were higher in patients treated with IS treatment, although this was not statistically significant (*p* Chi^2^ 0.5258).

Cause of Mortality	Nintedanib with IS Treatment (n)	Nintedanib Without IS Treatment (n)
<12 months after nintedanib initiation	AEx (1) Fibrosis Progression (2)	AEx (2) Neoplasia (2)
>12 months after nintedanib initiation	AEx (4) Neoplasia (2) Fibrosis Progression (3)	AEx (1) Neoplasia (1)

**Table 5:** Immunosuppressant treatment and ruxolitinib administered the year previous and subsequent to nintedanib treatment. Reductions in corticosteroid dose and immunosuppressant treatment were observed the year after nintedanib initiation without statistical analysis because of the small sample.

Immunosuppressant Treatment, n (%)	Previous Year Before Nintedanib Treatment n 42 from 55	The Year After Nintedanib Treatment n 38 from 55
Corticosteroids ≥15 mg/day 14–6 mg/day ≤5 mg/day Mycophenolate Rituximab Leflunomide Hydroxychloroquine Abatacept Methotrexate Tocilizumab Adalimumab Tacrolimus Sirolimus Azathioprine Ruxolitinib	40 (72.7%) 6 (10.9%) 17 (30.9%) 17 (30.9%) 13 (23.6%) 9 (16.4%) 4 (7.3%) 2 (3.6%) 2 (3.6%) 1 (1.8%) 1 (1.8%) 1 (1.8%) 1 (1.8%) 1 (1.8%) 0 (0%) 2 (3.6%)	33 (60%) 2 (3.6%) 8 (14.5%) 23 (41.8%) 14 (25.5%) 6 (10.9%) 2 (3.6%) 1 (1.8%) 1 (1.8%) 0 (0%) 2 (3.6%) 0 (0%) 1 (1.8%) 0 (0%) 1 (1.8%) 2 (3.6%)

## Data Availability

The data from this study are not publicly available owing to patient confidentiality and privacy. No new data were created or analysed in this study. Data sharing is not applicable to this article.

## Abbreviations

The following abbreviations are used in this manuscript:

CI	Confidence Interval
CTD-ILD	Connective tissue disease associated with interstitial lung disease
DLCO	Diffusing lung capacity for carbon monoxide
FVC	Forced Vital Capacity
fHP	Fibrotic Hypersensitivity Pneumonitis
HP	Hypersensitivity Pneumonitis
HRCT	High resolution computed tomography
ILD	Interstitial lung diseases
IPF	Idiopathic pulmonary fibrosis
IS	immunosuppressant treatment
n	sample size
NSIP	Non-specific interstitial pneumonia
Non-IPF	non-Idiopathic pulmonary fibrosis
*p*	*p*-value
PAH	pulmonary arterial hypertension
%	percentage
PPF	Progressive pulmonary fibrosis
Q1	Quartile 1
Q3	Quartile 3
SD	Standard deviation
6MWD	6 minute walking distance
6MWT	6 min walking test
SRIF	Smoking related interstitial fibrosis
SmPC	Summary of product characteristics
uILD	Unclassifiable interstitial lung disease
